# Comparison of Lateral and Crossed K-wires for Paediatric Supracondylar Fractures: A Retrospective Cohort Study

**DOI:** 10.7759/cureus.27267

**Published:** 2022-07-26

**Authors:** Bakhat Yawar, Mohammad Noah Khan, Ayeisha Asim, Ammal Qureshi, Ali Yawar, Ahmad Faraz, Andrew McAdam, Sami Mustafa, Brian Hanratty

**Affiliations:** 1 General Surgery, The Western Trust Health & Social Care Jobs in Northern Ireland (HSCNI) (Altnagelvin Area Hospital), Derry/Londonderry, GBR; 2 Trauma and Orthopaedics, Royal Victoria Hospital, Belfast, GBR; 3 Geriatrics, The Western Trust Health & Social Care Jobs in Northern Ireland (HSCNI) (Altnagelvin Area Hospital), Derry/Londonderry, GBR; 4 Trauma and Orthopaedics, The Western Trust Health & Social Care Jobs in Northern Ireland (HSCNI) (Altnagelvin Area Hospital), Derry/Londonderry, GBR; 5 Trauma and Orthopaedics, Newcastle upon Tyne Hospitals NHS Foundation Trust, Newcastle, GBR; 6 Urology, The Western Trust Health & Social Care Jobs in Northern Ireland (HSCNI) (Altnagelvin Area Hospital), Derry/Londonderry, GBR

**Keywords:** lateral capitellohumeral angle, baumann angle, paediatric orthopedics, elbow trauma, supracondylar humeral fracture

## Abstract

Background

Supracondylar elbow fractures occur most frequently in children aged five to seven years and have equal incidence in both genders. They are classified as flexion or extension type injuries with extension type being more common. We aimed to ascertain radiological stability with lateral and crossed wires in this study. We also identified any complications after operative management of these injuries.

Methods

As part of this retrospective cohort study, we identified all patients who presented with this injury from January 1, 2020, until February 28, 2022. Basic demographic data and type of operation were noted. Baumann angle (BA) and lateral capitellohumeral angle (LCHA) were measured intra-operatively and x-rays were done at the final clinic appointment. The mean of these angles in lateral and crossed wire groups was compared using paired sample t-test. Unpaired t-test was used to compare the means of both groups with normal values for these angles based on previous studies (BA=71.5±6.2 degrees, LCHA= 50.8±6 degrees).

Results

Fifty patients were admitted during this period. Thirty-three patients had lateral wires and 17 had crossed wires for fixation. No significant change was noted in the mean BA and mean LCHA in both groups on x-rays done intra-operatively and final clinic follow-up (no loss of reduction). No significant difference was noted between BA and LCHA noted for both groups at the final clinic follow-up with previous studies outlining normal values for these angles. No cases of iatrogenic neurovascular injury were identified. Four patients (8%) were referred to physiotherapy due to stiffness.

Conclusion

Both lateral and crossed wire configurations led to achievement of good radiological stability with BA and LCHA within normal limits. No loss of reduction was noted with both techniques and no risk of iatrogenic nerve injuries was noted in experienced hands.

## Introduction

Supracondylar elbow fractures are a common injury in the paediatric population and account for 60% of elbow fractures in this demographic [1]. Skeletally immature individuals between the age of five to 10 years are most likely to sustain this injury with peak incidence around six years of age [1]. Injuries can be classified as extension-type and flexion-type injuries [1]. Extension-type injuries constitute 95% of supracondylar fractures [1]. Further classification of extension-type injuries was first described by Gartland in 1963 who classified the injuries into three grades and defined treatment algorithms based on displacement of the fracture [2]. The classification system was further modified by Wilkins who added concepts of posterior humeral contact and rotational deformity [3]. Leitch et al. further proposed addition of grade 4 injuries which are defined as completely displaced fractures with multidirectional instability and are innately more difficult to treat [4]. Extension-type supracondylar fractures usually result from a fall onto the outstretched hand whereas flexion-type injuries are most likely to result from a direct blow to the posterior aspect of the flexed elbow [1].

Closed reduction and percutaneous pinning with K-wires is the treatment of choice for grade 2 and above supracondylar fractures. Two or three K-wires can be inserted as all lateral or crossed configuration. Crossed K-wires are reported to have greater rotational rigidity compared to lateral pins but have been reported be more likely associated with ulnar nerve injuries [5-7].

The primary aim of our study was to compare the radiological stability of supracondylar fractures treated with lateral and crossed K-wires using Baumann angle (BA) and lateral capitellohumeral angle (LCHA) measured intra-operatively and at the final clinic follow-up. In addition, we secondarily assessed patient demographics, common mechanisms of injury, duration of clinic follow-up, and post-operative complications in both groups.

## Materials and methods

Study design

This was a retrospective cohort study where we obtained data regarding all paediatric patients admitted with supracondylar elbow fractures from January 1, 2020, until February 28, 2022. Data were collected on a secure Microsoft Excel sheet. This study was classified as an audit and permission from the local audit and quality improvement department at Altnagelvin Area Hospital, Derry/Londonderry. A formal ethics approval or institutional review board approval was not required as this was deemed a retrospective cohort study in line with recommendations from National Health Service Health Research Authority (NHS HRA) regulations.

Inclusion and exclusion criteria

Paediatric patients between the age of two to 12 years presenting with supracondylar elbow fractures who were surgically managed were included in the study. Children managed non-operatively were excluded from the study. Also, we excluded children with other elbow fractures such as lateral or medial epicondyle fractures, olecranon fractures, and those requiring open reduction and internal fixation. We also excluded patients for whom further clinic follow-up was planned at the time of data collection.

Data collection

The local theatre management system (TMS) coordinators provided a list of patients who fit the inclusion criteria. Demographics, admission, and follow-up data were obtained from the patient records available through the Northern Ireland Electronic Care Record (NIECR). The Northern Ireland Picture Archiving and Communication System (NIPACS) was used to obtain accurate measurements of the angles used to assess fracture stabilisation.

Definitions

**Figure 1:**
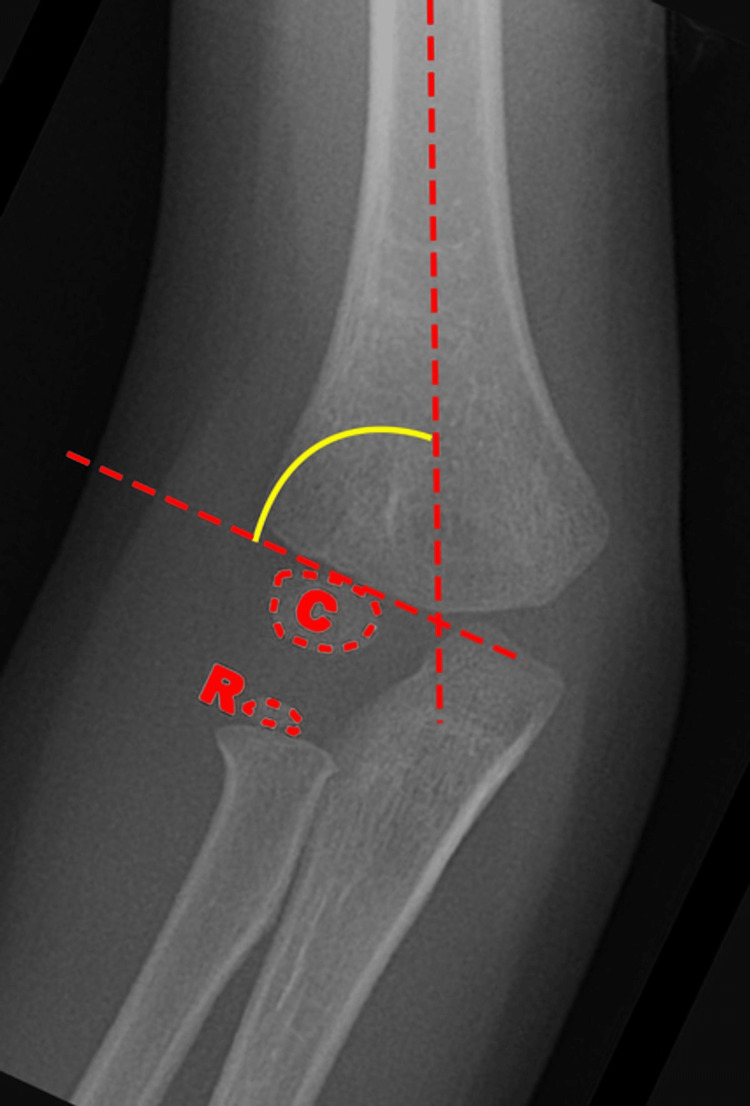
Illustration of Baumann angle (BA) which is denoted by the yellow curved line The image is taken from Benoudina and Weerakkody (2021) [13]; permission of use obtained. C: capitellum; R: radial head

**Figure 2:**
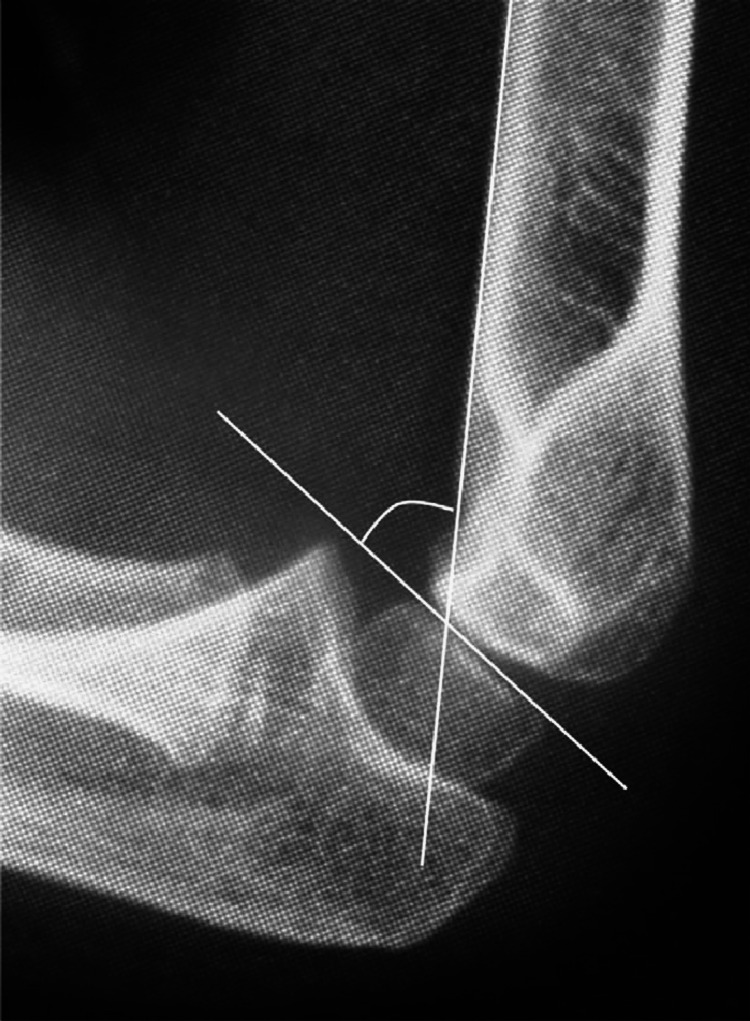
Illustration of lateral capitellohumeral angle (LCHA) The image is taken from Hasegawa et al. (2021) [14]; permission of use obtained.

Baumann angle (BA), also known as the capitellohumeral angle, is formed by the humeral axis and a line drawn through the epiphyseal plate of the capitellum. It is a measure of coronal plane alignment in supracondylar fractures. The lateral capitellohumeral angle (LCHA) is the angle between humeral shaft and the capitellum on lateral x-rays of the elbow and is a measure of the sagittal plane alignment [8]. The normal values of these angles were noted in several previous publications [9-12]. We used the values provided by Shank et al. as reference values for comparison with the BA and LCHA on final clinical x-rays on our patient population as this was a good study that provided mean BA and LCHA in normal uninjured children along with standard deviation [9]. The mean BA in this study was 71.5 degrees with a standard deviation of 6.2 degrees. The mean LCHA in this study was 50.8 degrees with standard deviation of 6 degrees. Figures 1, 2 below have been obtained from other sources which illustrate the BA and LCHA [13,14].

Fractures were classified as flexion-type and extension-type. Extension-type fractures were further classified according to modified Gartland classification as per Wilkins and Leitch et al. (Table 1) [3,4,15].

**Table 1:** Extension type fractures classified according to modified Gartland classification The table is adapted from Alton et al. (2015) [3], Leitch et al. (2006) [4], and Benoudina and Weerakkody (2021) [13].

Grade	Description
Grade 1	Fractures with no or minimal posterior displacement or angulation of the distal fragment such that the anterior humeral line still intersects part of the capitellum
Grade 2	Fractures with more posterior displacement or angulation, but with an intact posterior cortex; grade 2 fractures have been divided into grade 2A, with no rotation or translation, and grade 2B, with some rotation or translation in addition to posterior displacement and angulation
Grade 3	Fractures with displacement and complete cortical disruption
Grade 4	Fractures with displacement, complete cortical disruption, and complete loss of the periosteal hinge anteriorly and posteriorly leading to multidirectional instability

For convenience, grade 2 fractures were not further classified as part of the study. The mechanism of injury was classified as per Table 2. The rationale for such a classification system was to emphasise commonly associated hazardous activities resulting in upper limb fractures in children.

**Table 2:** Classification of mechanisms of injury

S. no.	Mechanism of injury
1.	Fall from climbing frame
2.	Fall from trampoline
3.	Fall from bouncy castle
4.	Fall from slide
5.	Other fall related mechanism of injury
6.	Other mechanism of injury

Data variables collected

Basic demographic variables included gender and age. Laterality of the fracture was also noted. The mechanism of injury was recorded as per Table 2 above. The type of fracture was noted and classified according to Table 1 above. Characteristics of injury noted included presence of nerve injury (including distribution of the nerve such as ulnar, median, anterior interosseous, and radial nerves), vascular injury, and soft tissue characteristics (i.e., open or closed injury). Additionally, we noted time from diagnosis (time of first x-ray confirming fracture) to time to operation in hours. In our department, both lateral and crossed wire configurations are employed for fixation of these fractures as per surgeon preference. Surgical management was mostly provided to patients with unstable grade 2 or above injuries and the decisions to operate were consultant-led and discussed in daily morning trauma meetings among senior consultants.

Configuration of K-wires was noted on intra-operative x-rays as all lateral versus crossed wires. K-wires of 2 mm were used in our centre to fix these injuries. Additionally, we recorded the number of wires inserted for stabilisation of the fracture. We also noted number of clinic follow-ups required, number of days to removal of K-wires, and number of days till removal of cast.

Operative complications noted included iatrogenic neurovascular injury, pin site infection, and presence of significant stiffness requiring referral to physiotherapy services. BA and LCHA were measured on intra-operative x-rays and x-rays taken at the final clinic follow-up.

Data analysis

As part of achieving our primary aim for this study, we used Statistical Package for the Social Sciences (SPSS) version 25 (Armonk, NY: IBM Corp.) to compare mean BA and LCHA at time of operation; and mean BA and LCHA at final clinic appointment in groups treated with lateral or crossed K-wires using paired t-test to ascertain the comparison between stabilisation afforded by both the techniques. We also performed a chi-square test to identify an association between configuration of wires and number of wires used.

As part of our secondary aims, we used an unpaired t-test to compare mean BA and LCHA at the final clinic appointment in both lateral and crossed wire groups with normal values for these angles as per Shank et al. [9]. We compared the mean number of days to removal of cast and the mean number of days till removal of wires in both crossed and lateral wire groups using unpaired t-test. We used odds ratio analysis to compare the odds of requiring physiotherapy due to significant post-operative stiffness in lateral wire versus crossed wire group.

## Results

Demographic and baseline characteristics

Baseline characteristics and demographic data are listed in Table 3 below. The groups of patients treated with lateral versus crossed wires were similar in terms of age and gender. Distribution in terms of mechanism of injury was also somewhat similar in both groups.

**Table 3:** Baseline characteristics of patients treated operatively for supracondylar elbow fractures SD: standard deviation: pre-op: pre-operatively

Variable	Category	All patients		Lateral wires patients		Crossed wire patients	
		N= 50	Summary	N=33	Summary	N=17	Summary
Age (mean±SD)	-	50	6.34±2.40	33	6.21± 2.53	17	6.38±2.19
Gender	Male	50	26 (52%)	33	20 (60.6%)	17	6 (35.3%)
	Female		24 (48%)		13 (39.4%)		11 (64.7%)
Mechanism of injury	Fall from climbing frame	50	6 (12%)	33	4 (12%)	17	2 (12%)
	Fall from bouncy castle		3 (6%)		1 (3%)		2 (12%)
	Fall from trampoline		8 (16%)		5 (15%)		3 (17%)
	Fall from slide		5 (10%)		4 (12%)		1 (6%)
	Other fall-related mechanism of injury		23 (46%)		16 (49%)		7 (41%)
	Other mechanism of injury		5 (10 %)		3 (9%)		2 (12%)
Laterality	Right	50	28 (56%)	33	18 (54%)	17	10 (59%)
	Left		22 (44%)		15 (46%)		7 (41%)
Type of injury	Flexion type	50	3 (6%)	33	3 (9%)	17	0 (0%)
	Grade 1		0 (0%)		0 (0%)		0 (0%)
	Grade 2		16 (32%)		15 (45.5%)		1 (6%)
	Grade 3		30 (60%)		15 (45.5%)		15 (88%)
	Grade 4		1 (2%)		0 (0%)		1 (6%)
Nerve injury pre-op	Yes	50	2 (4%)	33	2 (6%)	17	0 (0%)
	No		48 (96%)		31 (94%)		17 (100%)
Vascular injury pre-op	Yes	50	2 (4%)	33	0 (0%)	17	2 (12%)
	No		48 (96%)		33 (100%)		15 (88%)
Number of wires used	2	50	30 (60%)	33	26 (79%)	17	4 (24%)
	3		20 (40%)		7 (21%)		13 (76%)

A chi-square test was performed to ascertain an association between configuration of wires and number of wires used. The relation between these variables was significant χ2 (1, N=50) =14.2751, p<0.001. Patients treated with crossed K-wires were more likely to have three K-wires inserted. Mean time to surgery from the initial x-ray was 16.04 hours with a standard deviation of 10.97 hours (range= 2-58 hours).

Stabilisation of fracture (primary aim)

The mean BA and LCHA measured intra-operatively and at time of final clinic follow-up did not vary significantly in both the groups treated with crossed and lateral wires. This would suggest that the fractures treated with both lateral and crossed wires maintained stability throughout the period of fracture healing and follow-up. Table 4 below provides useful information for explanations.

**Table 4:** Comparison of BA and LCHA between intra-operative values and final clinic x-ray values BA: Baumann angle; LCHA: lateral capitellohumeral angle

Outcome	Category	Number of patients (N)	Mean ± SD (degrees)	t- Statistic	p-Value
BA in lateral wire group	Intra-operative	33	69.2±2.1	0.89	0.381
	Final clinic	33	69.7±3.0		
LCHA in lateral wire group	Intra-operative	33	51.4±6.5	0.45	0.652
	Final clinic	33	51.7±5.8		
BA in crossed wire group	Intra-operative	17	69.8±2.3	0.22	0.829
	Final clinic	17	69.9±3.2		
LCHA in crossed wire group	Intra-operative	17	53.1±4.3	0.18	0.095
	Final clinic	17	54.6±5.2		

Fracture fixation compared to normal values of BA and LCHA

Our study found no significant difference between the mean BA and LCHA measured on x-rays at final clinic follow-up compared to the normal values in uninjured elbows as per Shank et al. [9]. The only significant difference was for comparison of LCHA in patients treated with crossed wires and normal values of the LCHA. LCHA was found to be higher in crossed wire group. This would suggest that adequately managed supracondylar fractures intra-operatively in terms of reduction intra-operatively provide good radiological results with both crossed and lateral wires in terms of BA. Table 5 below provides useful information for explanation.

**Table 5:** Final BA and LCHA compared to normal values of the angles BA: Baumann angle; LCHA: lateral capitellohumeral angle

Outcome	Category	Number of patients (N)	Mean ± SD (degrees)	t- Statistic	p-Value
Comparison of BA for lateral wire group	Lateral wire group	33	69.7±3.0	1.58	0.12
	Reference values	71	71.5±6.2		
Comparison of LCHA for lateral wire group	Lateral wire group	33	51.7±5.8	0.72	0.47
	Reference values	71	50.8±6		
Comparison of BA for crossed wire group	Crossed wire group	17	69.9±3.2	1.03	0.31
	Reference values	71	71.5±6.2		
Comparison of LCHA for crossed wire group	Crossed wire group	17	54.6±5.2	2.39	0.02
	Reference values	71	50.8±6		

Follow-up outcome data

The odds of having significant stiffness requiring referral to physiotherapy were greater in crossed wire group compared to lateral wire group (OR=6.86) but this was not statistically significant (p=0.1081). There was no significant difference noted in the comparison of mean time to removal of wires in lateral versus crossed wire groups. The time to removal of cast and mobilisation was noted to be significantly longer in crossed wire group. This is further elaborated in Table 6 below.

**Table 6:** Comparison of follow-up outcomes in patients treated with lateral and crossed wires

Outcome	Category	Number of patients (N)	Mean ± SD (days)	t- Statistic	p-Value
Time to removal of wires (days)	Lateral wire group	33	25.39±4.12	0.35	0.72
	Crossed wire group	17	24.94±4.44		
Time to removal of cast (days)	Lateral wire group	33	26.24±4.65	2.40	0.02
	Crossed wire group	17	30.82±8.89		

## Discussion

Supracondylar humeral fractures are commonly seen in children, and they are reported to be the second most common injury in children [16]. Our data show a mean age of around six years with almost equal distribution in terms of gender which is in line with demographic data reported for this study [1]. A vast majority of patients sustained the injury during recreational activities and among our recorded mechanisms of injury fall from trampolines and climbing frames were major contributing factors. Some previous literature has advocated the introduction of parent education, parental supervision, and safety measures such as use of rubber surfacing in playgrounds to reduce the incidence of such injuries [17-20]. Four percent patients had a neurological deficit as a result of the fracture, and all of these were neurapraxias that resolved with conservative management for the nerve injury. Most nerve injuries have been described to be neurapraxias in setting of supracondylar fractures in previous literature [19]. Vascular compromise was noted in 4% patients in our study. The incidence we noted was lower as compared to previously reported figures. With reduction of the fracture, vascular supply was restored in all our patients. Previous studies also describe that most cases of vascular compromise associated with supracondylar fractures resolve with fracture reduction [21,22]. The British Orthopaedics Association (BOA) has developed standards for management of supracondylar humeral fractures in children and recommends the use of 2mm K-wires for stabilisation of the fracture. The guidelines also set standards for appropriate neurovascular assessment and management of neurovascular injuries associated with these fractures [23]. These guidelines are the cornerstone of the protocol for management of supracondylar fractures in our hospital system.

We noted in our study that the mean BA and LCHA at time of final X-rays were not statistically significant compared to mean BA and LCHA measurements taken intra-operatively after fixation of fracture. Previously it has been reported that higher degree of torsional stress is needed to deform fractures fixed with crossed wires compared to lateral wires, but this remains a controversial topic [24]. Other studies showed no difference in stability provided by the two types of techniques [25]. Our study showed that practically there is no significant difference between the techniques in terms of maintaining fracture stability. We chose the study by Shank et al. because this was the only study that described the values of both BA and LCHA in normal uninjured elbows [9]. Our study showed that there was a significant difference between the mean LCHA measured at final clinic appointment in patients managed with crossed wires in our study and the normal LCHA described by our reference study. We believe the reason may be because more patients with severe injury (grade 3 or grade 4 injuries) were managed with crossed wires and reduction itself may have been more difficult in these patients compared to patients managed with lateral wires. Incidence of iatrogenic ulnar nerve injury has been described to be between 4-15% in literature [26-28]. In our study, no iatrogenic ulnar nerve injury was noted with any type of technique used for fracture fixation. The time to removal of cast was longer in the group managed with crossed wire fixation. This may also be explained by the greater severity of injury noted in patients treated with crossed wires. Of note, a greater proportion of patients with grade 3 or grade 4 injuries were managed with crossed K-wiring.

Our study had limitations in terms of a small number of patients due to which some data did not reach statistical significance such as the presence of higher stiffness in patients managed with crossed wires leading to a greater need for referral to physiotherapy. Nevertheless, the study gives further evidence to the notion that excellent results can be achieved with both lateral and crossed wires in management of supracondylar elbow fractures in children even though we note that the BOAST guidelines report lower risk of loss of reduction with use of crossed wires [23]. We recommend use of both techniques dependent on consultant's preference in management of these injuries.

## Conclusions

Based on radiological follow-up, lateral and crossed K-wiring techniques for supracondylar elbow fractures lead to superb construct stiffness which in turn provides stability to the fracture site during the healing process. In experienced hands, the incidence of post-operative complications such as iatrogenic nerve injury is also low compared to previously available data.
